# Hydrocracking of Heavy Fischer–Tropsch Wax Distillation Residues and Its Blends with Vacuum Gas Oil Using Phonolite-Based Catalysts

**DOI:** 10.3390/molecules26237172

**Published:** 2021-11-26

**Authors:** Jakub Frątczak, Héctor de Paz Carmona, Zdeněk Tišler, José M. Hidalgo Herrador, Zahra Gholami

**Affiliations:** ORLEN UniCRE, a.s., Revoluční 1521/84, 400 01 Ústí nad Labem, Czech Republic; Hector.Carmona@unicre.cz (H.d.P.C.); Zdenek.Tisler@unicre.cz (Z.T.); jose.hidalgo@unicre.cz (J.M.H.H.); Zahra.Gholami@unicre.cz (Z.G.)

**Keywords:** hydrocracking, co-processing, phonolite, Fischer–Tropsch wax

## Abstract

The Fischer–Tropsch heavy fraction is a potential feedstock for transport-fuels production through co-processing with fossil fuel fraction. However, there is still the need of developing new and green catalytic materials able to process this feedstock into valuable outputs. The present work studies the co-hydrocracking of the Fisher–Tropsch heavy fraction (FT-res.) with vacuum gas oil (VGO) at different ratios (FT-res. 9:1 VGO, FT-res. 7:3 VGO, and FT-res. 5:5 VGO) using phonolite-based catalysts (5Ni10W/Ph, 5Ni10Mo/Ph, and 5Co10Mo/Ph), paying attention to the overall conversion, yield, and selectivity of the products and properties. The co-processing experiments were carried out in an autoclave reactor at 450 °C, under 50 bars for 1 and 2 h. The phonolite-based catalysts were active in the hydrocracking of FT-res.:VGO mixtures, presenting different yields to gasoline, diesel, and jet fuel fractions, depending on the time of reaction and type of catalyst. Our results enable us to define the most suitable metal transition composition for the phonolite-based support as a hydrocracking catalyst.

## 1. Introduction

Fossil fuel consumption is one of the most significant causes of global warming and climate change. The EU’s target for 2030 is to reduce greenhouse gas (GHG) by 50–55%, compared with the 1990 levels, and to achieve net-zero GHG emissions by 2050 [1]. The most significant CO_2_ emissions come from the transportation sector (30% in 2020), and a further decrease in CO_2_ emissions from other sectors, it is expected to reach approximately 38% in 2050. With approximately 71% of emissions in 2018, road transport has the highest portion of the GHG emissions among the transport sector in the European Union (EU), and it is must be reduced in the future [2]. Limited fossil fuel sources, the increasing demand for energy resources, and the environmental restrictions and rules regarding fossil fuel utilization have led to new approaches to producing renewable and clean energy.

Different XTL (X to liquid fuels) technologies, including natural gas (GTL), coal (CTL), biomass (BTL), and waste/oil residues (WTL), have been used for the conversion of carbon-containing sources to liquid fuels [3,4]. Through these technologies, the carbonaceous feedstock, from different sources such as coal, natural gas, or biomass, first transforms into syngas (CO + H_2_). Then, it converts to a wide range of hydrocarbons, which are refined to obtain the desired products such as liquefied petroleum gas (LPG), gasoline, jet fuel, diesel, and wax. [5]. Fischer–Tropsch(FT) synthesis is one of the most well-known processes for the catalytic conversion of syngas to a wide range of products, such as paraffins, olefins, alcohols, and aldehydes, which can be then upgraded to aromatic- and sulfur-free fuels and chemicals [6,7,8]. According to their properties, FT products are categorized into gaseous hydrocarbons (C_1_–C_4_), light oxygenated (C_1_–C_5_), naphtha (C_5_–C_10_) (with a boiling range of 30–175 °C), distillate (C_11_–C_22_) (with a boiling range of 175–340 °C), and atmospheric residue, which is either a wax or an aromatic-rich oil with a boiling point of higher than 340 °C [9]. The kerosene fraction, with the boiling point range of 160–260 °C, is used to produce jet fuel, and it overlaps with both distillate and naphtha fractions. The atmospheric residue can be separated into two categories of vacuum residual oil (VR), and vacuum gas oil (VGO) through vacuum distillation [10]. VGO is usually used as the feedstock in cracking units to be upgraded to gasoline and diesel. Moreover, it can be used as a petroleum blending component for the co-processing mixtures. Generally, the heavy hydrocarbon products of the FT process need to be cracked into middle distillate range hydrocarbons [11].

Hydrocracking is an exothermic catalytic refining process. It is used to upgrade heavy hydrocarbons, such as FT wax, VGO, and heavy gas oil, to lighter products such as naphtha, kerosene, and diesel. During this process, olefins and oxygenates can also be converted to paraffins. Hydrocracking catalysts comprise metals and acidic support, whereas the active metal sites promote the hydrogenation/dehydrogenation of the paraffins, and the cracking function is provided by acidic support [12]. The ratio between the cracking and hydrogenation functions of the catalysts (i.e., acidic support and active metals) as an essential parameter that can be adjusted to reach the optimum catalytic performance. The higher hydrogenation activity results in heavier and less isomerized products, while the higher acidity leads to higher activities, higher isomerization degrees, and lighter product slates. The catalyst can be selected based on the required properties of the products. Both noble metals (e.g., Pt, or Pd) and non-noble metals (e.g., Co/Mo, Ni/Mo, Ni/W, or Co/W) can be used as an active metal for the cracking reaction. Amorphous carriers can be used when the maximum production of middle distillate is desired.

In contrast, a mixture of amorphous/zeolite carriers can lead to the formation of more kerosene and naphtha. The most used amorphous carrier in the commercial hydrocracking catalysts is silica-alumina, and Zeolite Y is also regularly used as a binder with silica-alumina or alumina [13]. Phonolite, comprising mainly alkali feldspar, is an inexpensive, readily available compound, and can be used as a potential support material [14]. After acid treatment, the solid non-porous phonolite becomes a highly porous material with a significant surface area. This acid-treated phonolite can be used as support for catalysts. A series of NiW catalysts supported on acid-modified phonolite, foamed zeolites, and alumina were used for the deoxygenation of waste rendering fat [15]. The phonolite-supported catalyst showed a high activity in the deoxygenation reaction. Moreover, this catalyst had the lowest deposited carbon on the surface, which could enhance the catalyst lifetime and decrease the deactivation possibility.

The quality of the cracked products is affected by the type of catalyst and the processing conditions. Production of fuel range hydrocarbons via hydrocracking of FT wax and atmospheric residues has been widely investigated [16,17,18,19,20,21,22]. High cetane numbers of fuels help reduce particle matters, NO_x_, CO, and hydrocarbon emissions. Cloud points of approximately −15 °C are generally suitable for winter-grade diesel fuels. A combination of low sulfur and aromatics content, high cetane numbers (>52), and low cloud points are the desired properties of the produced fuel range hydrocarbons. Leckel [17] studied the low-pressure hydrocracking of FT wax for diesel production using sulfided NiMo/SiO_2_-Al_2_O_3_, NiW/SiO_2_-Al_2_O_3_, and a non-sulfidic noble metal catalyst modified with MoO_3_ (Pt/MoO_3_/SiO_2_-Al_2_O_3_). All catalysts were suitable for diesel fuel production with high cetane numbers (60–73) and low cloud points (−12 °C to −28 °C). The cracking conversion increased by approximately 30% by lowering the pressure from 7.0 MPa to 3.5 MPa. The produced diesel fuel also had meagre contents of aromatic (<0.5%) and sulfur (<5 ppm).

Hydrocracking of a mixture of 10 wt.% FT wax and 90 wt.% VGO was investigated by Halmenschlager et al. [18]. The results revealed that the atmospheric residue conversion of 100% VGO was higher than that of the mixture of VGO and wax during the hydrocracking reaction at 9.5 MPa and 330–410 °C, over a sulfided NiMo/SiO_2_-Al_2_O_3_ catalyst. The solubility parameter of the bulk liquid decreased by the addition of wax to VGO, which can alter the relative solubility of the heavier and more polar species in VGO, resulting in different surface compositions of VGO species, and the number of species that are more resistant towards hydrocracking. It is necessary to have a catalyst with high activity for the hydrocracking of both wax and VGO, which can be achieved by increasing the acid strength of the support materials such as zeolites with large pore sizes.

In this way, a commercial zeolite-supported catalyst was used for the hydrocracking of a mixture of bitumen-derived hydrotreated heavy vacuum gas oil (HVGO) and FT wax in an autoclave reactor at 360 °C and 4.2 MPa [21]. The complete FT wax conversion was obtained when the content of FT wax in the mixture was more than 50 wt.%, while at the lower content FT wax, the FT wax conversion was not complete. This behavior could be attributed to the higher adsorption of the polar components (aromatics and heteroatoms) in HVGO, rather than paraffins in the wax. By increasing the content of FT wax in the feedstock mixture, the octane number of the naphtha fraction also increased. At the same time, the contents of cycloparaffins and aromatics increased by increasing the FT content in the feedstock. In another research by Šimáček et al. [23], co-processing of a mixture containing 10 wt.% of FT wax and 90 wt.% of the petroleum-derived vacuum distillate (VGO) was performed in a fixed-bed reactor at 18 MPa and the temperature in the range of 390–430 °C over a commercial Ni–W/alumina catalyst. The produced diesel fraction from the hydrocracking of the mixture of VGO and FT wax had a cetane number 4–6 times higher than the diesel fraction obtained from the hydrocracking of VGO only. The cetane number of 58 and cloud point of −18 °C were observed in the diesel fraction of the co-processed mixture at 430 °C. In their recent study, Pleyer et al. [24] showed that the yield of gaseous products is increased by an increase in the content of the FT wax in the feedstock (HVGO + FT wax), while the yield of liquid products decreased at the same time.

In this paper, the hydrocracking of a mixture composed by VGO with different ratios of FT wax was investigated in an autoclave reactor at 450 °C, and under 50 bar over a series of phonolite (Ph) supported Ni-W (5Ni10W/Ph), Ni-Mo (5Ni10Mo/Ph), and Co-Mo (5Co10Mo/Ph) catalysts. The effect of FT wax content on the overall conversion, yield, and selectivity of the products, including diesel, gasoline, and jet fuel, and their properties and compositions are evaluated for the different catalysts.

## 2. Results and Discussion

### 2.1. Evaluation of the Hydrocracking Products

The yields of the products after hydrocracking reaction at different reaction conditions are shown in Table 1. The highest yield of the liquid products (71%) was obtained using the 5Ni10Mo/A-Ph catalyst after 60 min of reaction at 450 °C and the feedstock containing 50% VGO, which had been decreased to 59% by increasing the reaction time to 120 min. This phenomenon suggests that a longer reaction time led to secondary cracking reactions, improving the gasification and polymerization, which seems to be confirmed by an increment in the gas amount produced by this reaction (21%) and total solids (21%). In addition, the deactivation process could generate carbonaceous species, which could be deposited on the metal sites and/or support of the catalyst affecting the final yields to liquids. After adding 10% VGO to the feedstock, the yield of liquid products decreased in all reactions. The formation of liquid products increased by increasing the VGO content from 10% to 50% in the feedstock after 60 min of reaction using the 5Ni10Mo/A-Ph and 5Ni10W/A-Ph catalyst. For the 5Co10Mo/A-Ph catalyst, the content of liquid products did not change much by the feed composition; moreover, the liquid content for all reactions after 120 min was 54–61%, and did not change significantly by the variations in the feedstock composition. Xing et al. [21] reported that during the hydrocracking of the mixtures by FT wax/HVGO ratios of more than 50/50, an increase in the FT wax content led to the lower yield of liquid products. The surface of the sulfided catalyst is polar. Thus, polar molecules are expected to have a higher possibility for adsorption on the surface for molecules of equal size. The heterocyclic compounds are the most polar, and alkanes have the lowest polarity [18]. The presence of more polar species in the VGO could increase the hydrocracking conversion, and it could be the reason for increasing the higher yield of liquid products by increasing the VGO content from 10% to 50% in the feedstock. The solubility parameter of the polar compounds decreased at the higher FT residue content of the feedstock, which could increase the thermodynamic driving force for the material with higher solubility parameters (polarity, which improves the adsorption on a polar surface) to adsorb on the surface of the catalyst. The higher reactivity of the species with a higher polarity increases the overall solubility of the reactants (surfactant effect) parameters of VGO resulted in a higher feedstock conversion.

As written previously, the addition of VGO (polar and aromatic molecules) could impact the final yield of solids, affecting the overall kinetics of the hydrocracking. For the Ni-W catalyst, the addition of VGO did not show a clear trend for the gas or liquids production when the time of reaction was 1 h. Using a feedstock with a 90 wt.% of FT res., the solids content was higher than when the reaction time was 2 h, indicating that long molecules from FT waxes did not react to form shorter liquid organic molecules (solids at room temperature). A more significant difference was found for the reactions using 70 wt.% of FT res. from 1 h to 2 h of reaction time. However, at 2 h of reaction, the yields of solids, liquids, and gases were comparable. A similar result was found for the reaction using 50 wt.% of FT res. and 50 wt.% of VGO with a less significant decrease in the solid products comparing reactions with 1 and 2 h of reaction.

The reactions performed with the Ni-Mo catalyst led to similar results to the Ni-W catalyst tests. The exception was found for the reaction using 70 wt.% of FT res. with a significant increase for the solids at 1 h of reaction and the maximum yield of liquids for the reaction performed during 2 h. This particular reaction was similar to the reaction by using Ni-W catalyst with the same wt.%, indicating that this mixture of 70 wt.% of FT res. and 30 wt.% of VGO led to a minimum of liquids at 1 h of reaction. Nevertheless, at 2 h of reaction, the yield of liquids using Ni-Mo was the highest one. It seems that this specific mixture affected the reaction rate negatively, as shown for the tests performed during 1 h.

For tests using Co-Mo as the catalyst, similar yields were found for the reactions performed at 1 and 2 h. Thus, the reaction rate was not significantly affected by the content of FT res. or VGO. In this case, the maximum yield of liquids was found when pure FT res. was processed. However, to better understand the process, studying the results from the other analyses, such as the SIMDIS or heating value, is necessary because the produced molecules after hydrotreating and hydrocracking were different, depending on the used catalyst.

The heating values of the hydrocracking liquid products at different reaction conditions are also shown in Table 1. The heating value of the fuel is the standard method for the evaluation of its energy content. The amount of released heat after the complete burning of fuel is considered its heating value, and fuels with the higher heating values have the higher possible energy output [25]. The heating value can be reported based on the gross calorific value (higher heating value, HHV) and net calorific value (lower heating value, LHV) [26]. The gross and net calorific values of the hydrocracking products in this study did not significantly vary by changing the reaction conditions. They were in the range of 41.85–46.62 MJ/Kg and 39.27–43.50 MJ/kg for gross and net calorific values, respectively. The highest gross and net calorific values were observed for the product of reaction 11, where the 5Ni10Mo/A-Ph catalyst was used for the hydrocracking of the feedstock containing 10% VGO for 120 min. The lowest values belonged to reaction number 24. By adding 10% VGO to the feedstock, the heating value increased slightly. Then, it decreased by the further increase of VGO content in the feedstock, with the exception the reactions over 5Ni10Mo/A-Ph catalyst after 60 min where the lowest heating value observer for the feedstock with 10% VGO.

The heating value of the fuel can be estimated according to the fuel’s C, H, and O content, and its environmental impact can be determined based on the N and S contents [27]. Due to the high sulfur content in the VGO (19200 ppm), the sulfur content of the products also increased by increasing the VGO content (Table 2). Hydrogen has a heating value of 141.65 MJ/kg, which is the highest among the fuels (except for nuclear energies), and the higher hydrogen content results in a better combustion/ignition [28,29]. However, in this study (Table 2), the hydrogen content of the products is in the range of 12.8–14.6%, and did not change significantly in different reactions. However, the hydrogen concentration decreased slightly by increasing the VGO content in the feedstock, and the lowest concentration of 12.8% was observed for the product of reaction number 24.

**Figure 1:**
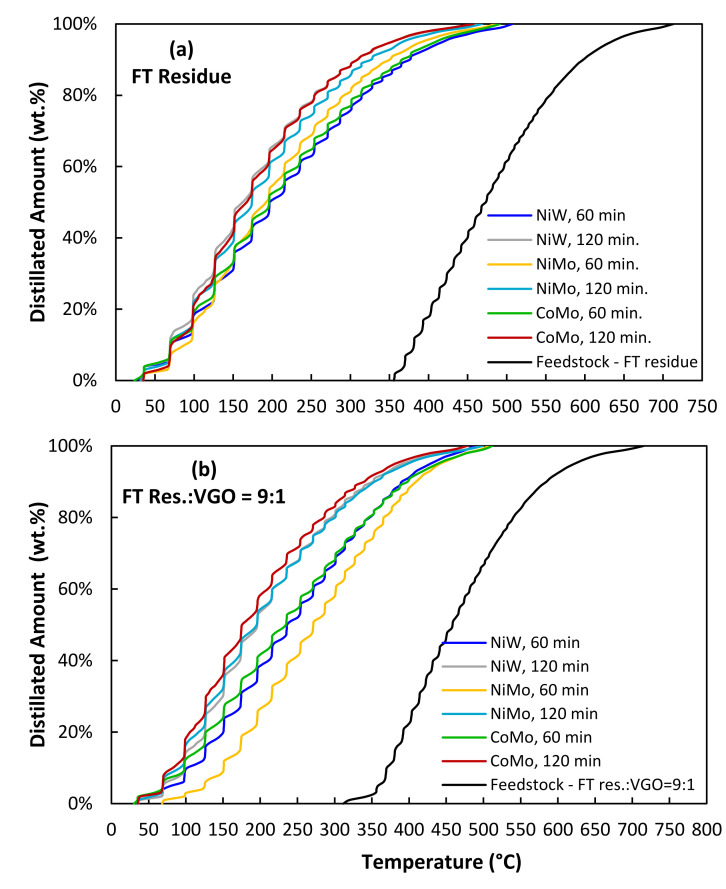

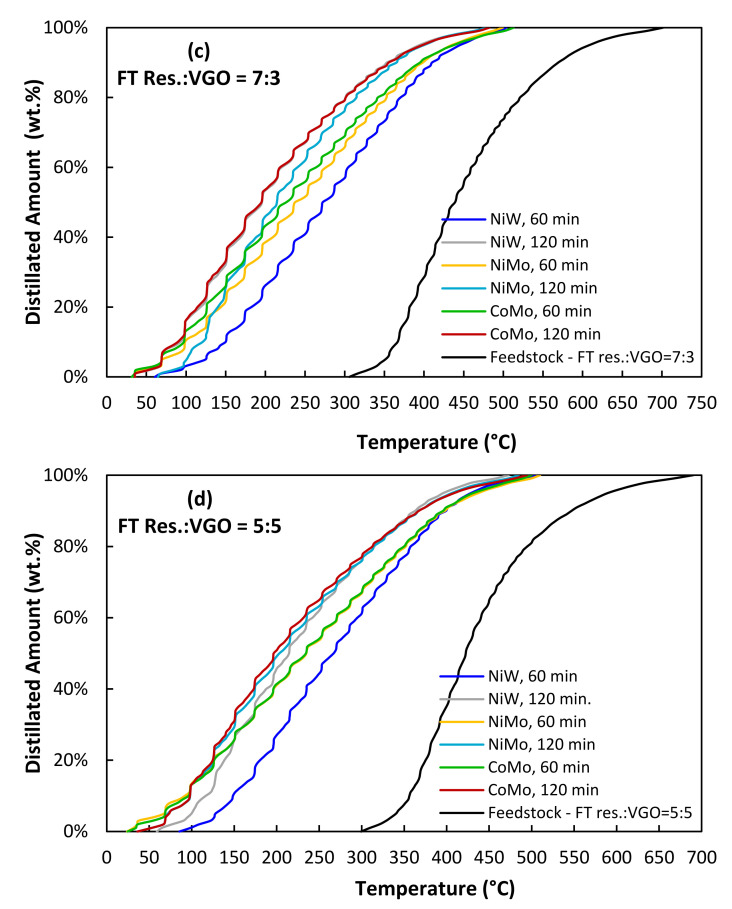
SIMDIS analysis of the feedstock and the products after hydrocracking reaction at different reaction conditions and different catalysts with different feedstock compositions: (**a**) FT residue, (**b**) FT Res.:VGO = 9:1, (**c**) FT Res.:VGO = 7:3, (**d**) FT Res.:VGO = 5:5.

The results presented in Table 2 show a linear relationship between the VGO content and density of the products, and the density increased by increasing the VGO content in the feedstock. The refractive index of the products also followed the same trend, and higher VGO content resulted in a higher refractive index. The product’s lower density and refractive index with higher FT residue content in the feedstock indicate lighter and more paraffinic products. The SIMDIS analysis of the hydrocracking products over different catalysts and different reaction conditions is shown in Figure 1. The lighter products with a lower boiling point than the feedstocks were obtained for all reaction conditions. The boiling point of the feedstocks was in the range of 300–700 °C, which decreased to less than 500 °C for the products. Similar to the variation of the density and refractive index, the boiling point of the products also increased by increasing the VGO content, and the lightest product with the lowest final boiling point (FBP) of 465 °C belonged to the products of FT residue hydrocracking over 5Ni10W/A-Ph catalyst after 120 min of reaction.

The highest number of compounds distilled at lower temperatures was found using a Co-Mo catalyst for the tests in almost all cases. The Ni-W tests results were different at the two different times of reaction. These results were in agreement with the mass balance studies. Ni-W tests at 1 h of reaction led to products with a higher boiling point range, and, at 2 h of reaction testing, the products’ boiling point ranges were much lower than the 1 h of reaction testing, which can suggest secondary cracking reactions. For Ni-Mo catalyst tests, the use of 10 wt.% of VGO led to the highest difference between the yields to lighter products (lower boiling points) using 1 and 2 h of reaction testing, compared to the other catalysts tests. In the Ni-Mo catalyst tests, the results were more similar to the results of the Co-Mo tests. Thus, these results demonstrate that the use of different VGO and FT res. wt.% ratios and the type of catalyst (Ni-W, Ni-Mo or Co-Mo) are essential factors to consider for obtaining different yields of products fraction. These results will help the current and future co-processing of FT waxes and VGO mixtures in the industry.

### 2.2. Composition of the Gaseous Products

**Figure 2:**
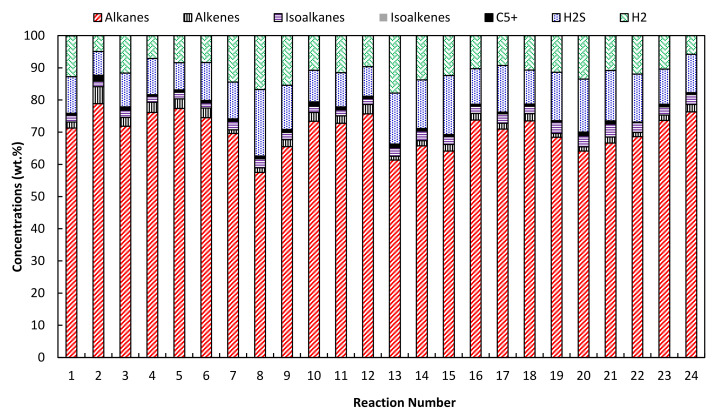
Composition of the gaseous products of the hydrocracking reaction.

The analysis of the gaseous products of the hydrocracking (Figure 2, Table 3) showed that alkanes are the main component of these products. The highest concentration of alkanes (C_1_–C_5_) was observed for reaction number 2 with 78.89 wt.%, where the FT residue went through the hydrocracking over 5Ni10W/A-Ph catalyst for 60 min. This reaction also had the lowest concentration of hydrogen and hydrogen sulfide. Adding 10% VGO to the feedstock decreased the alkane concentration, while the concentration of isoalkanes slightly increased. The concentration of H_2_ and H_2_S increased by approximately three times by the addition of 10% VGO to the FT residue wax. The higher VGO content (more than 10%) in the feedstock caused an increase in the alkanes and isoalkanes. The alkane formation was enhanced for all catalysts by increasing the reaction time from 60 min to 120 min. Hydrocracking of FT residue wax resulted in more linear paraffinic products, while the addition of VGO, with more aromatic and heteroatom contents, resulted in a slightly higher isoalkanes formation. Similar to the results obtained by Pleyer et al. [23], the higher FT residue content enhanced the yields of gaseous products, and increased the content of alkanes. The presence of C_5+_ hydrocarbons in the gaseous products could be due to some portion of these volatile components in the gas phase products during the hydrocracking. However, this study’s highest C_5+_ fraction in gaseous hydrocracking products is 1.64%, much lower than the portion of C_5+_ in the gaseous products (22–41%) observed by Pleyer et al. [23]. The significant loss of C_5+_ from the liquid phase can strongly affect the properties and composition of liquid products such as naphtha.

### 2.3. Hydrocracking Standard Products: Gasoline, Diesel, and Jet Fuel Fractions

The conversion, selectivity, and gasoline yield are measured according to equations 1, 2, and 7 (Table 4). The highest conversion of 84.18% was obtained for the hydrocracking of the feedstock containing 30% VGO using the 5Ni10W/A-Ph catalyst after 60 min of the reaction. Despite its highest conversion, this particular reaction (reaction number 13) had the lowest gasoline selectivity (4.64%) and yield (1.95%). The highest gasoline selectivity (51.96%) was observed for reaction number one, where the FT residue was used as the feedstock of hydrocracking over the 5Ni10W/A-Ph catalyst. Results revealed that the conversion was gradually increased by adding VGO up to 30%, and then by a further increase in the VGO content to 50%, conversion decreased. However, the gasoline selectivity and yield followed the opposite trend, and the highest selectivity to gasoline was obtained from the hydrocracking of FT residue. The highest yield of 17.13% was obtained for the hydrocracking of the FT residue over the 5Ni10W/A-Ph catalyst.

Similar to the gasoline fraction, the diesel fraction’s conversion, selectivity, and yield were calculated according to equations 3, 4, and 7 (Table 4). The highest conversion of 96.92% was observed for the hydrocracking of FT residue using the 5Co10Mo/A-Ph catalyst. The highest selectivity and yield of diesel were also observed for the hydrocracking of FT residue over the 5Ni10W/A-Ph catalyst. Similar to the gasoline fraction, the lowest selectivity and diesel yield was obtained for the reaction number 13.

Surprisingly, the conversion and selectivity of the jet fuel fraction did not follow the same trend as gasoline and diesel fractions (Table 4). The highest conversion of 92.75% was observed for the hydrocracking of the feedstock containing 30% VGO using the 5Ni10Mo/A-Ph catalyst, which also had the highest yield of jet fuel (21.41%). For the hydrocracking using the 5Ni10W/A-Ph catalyst, an increase in the VGO content (up to 30%) resulted in higher conversion, while the conversion decreased at the higher VGO content of 50%. The lowest observed selectivities and yields of gasoline, diesel, and jet fuel in reaction number 13 could be due to the lowest liquid product (18%) obtained over this reaction, much lower than those of other reactions (Table 1).

## 3. Materials and Methods

### 3.1. Materials

The sulfur-free synthetic FT wax produced by the Sasol Chemicals, Hamburg, Germany (Sasobit LM), and the VGO was supplied by a commercial refinery. The FT wax was sent to the fractional distillation to collect the desired amount of heavy FT distillation residue. The light fractions with boiling points of less than 360 °C were separated.

**Figure 3:**
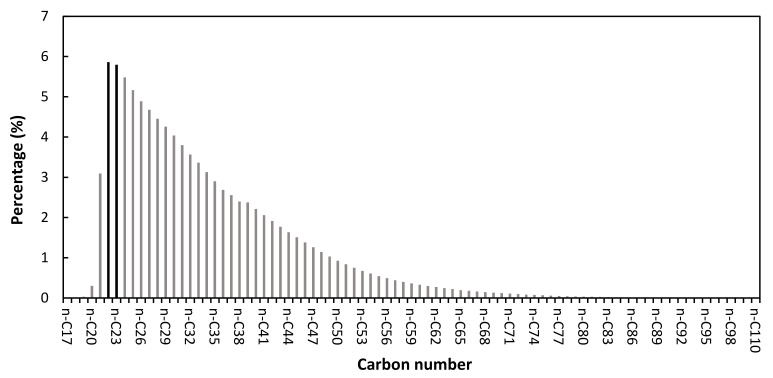
Distribution of hydrocarbons in FT residue.

This study used the heavy fractions of distillation residue as the FT residue (FT res.). Since the FT wax is a feedstock with low sulfur contents, and the catalysts should be activated in a sulfide form, three blends of FT residue and VGO, with different weight ratios of FT res.: VGO of 9:1, 7:3, and 5:5 were prepared. This fact allowed for increasing the sulfur level of the feedstock, maintaining the catalyst activity during the hydrocracking reaction, and studying the effect of the co-processing of these two feeds. The characteristics of the FT wax, FT residue, VGO, and their different blends are presented in Table 5. The hydrocarbon distributions of the FT residue are also shown in Figure 3. The highest concentrations were found for n-docosane (n-C_22_) with 5.86%, and n-tricosane (n-C_23_) with 5.80%.

The phonolite used as catalyst support was supplied by Keramost (Obrnice) [30] in the Czech Republic. The raw phonolite sand (224–560 μm) was dealuminated by an acid treatment using 3M hydrochloric acid, according to the method proposed by Hidalgo Herrador et al. [13]. The acid-treated phonolite (A-Ph) was then used as a support material for catalyst preparation. Three different types of catalysts, 5Ni10W/A-Ph (5wt.%Ni10wt.%W/A-Ph), 5Ni10Mo/A-Ph (5wt.%Ni10wt.%Mo/A-Ph), and 5wt.%Co10wt.%Mo/A-Ph (5wt%Co10wt%Mo/A-Ph), were prepared by co-impregnation of metal precursors (nickel/cobalt nitrate hexahydrate, ammonium heptamolybdate, and ammonium metatungstate) on phonolite, and the prepared catalysts were finally calcined in air for 6 h at 450 °C (1 °C/min).

The brief characteristics of utilized support material and synthesized catalysts is presented in Table 6.

Further analysis of the prepared catalysts, including scanning electron microscope (SEM), energy-dispersive X-ray spectroscopy (EDS), hydrogen temperature-programmed reduction (H_2_-TPR), and ammonia and CO_2_ temperature-programmed desorption (NH_3_-TPD, CO_2_-TPD), can be found in previous works from the authors [31,32].

### 3.2. Catalytic Hydrocracking

The hydrocracking reactions were carried out in an autoclave reactor (Parr Instruments, Moline, IL, USA, model 4575/76; 300 mL volume of reaction vessel) equipped with a “4848B” controller. Before the hydrocracking reaction, the catalysts were activated in the autoclave using a commercial sulfuring agent (Sulfurzol 54) supplied by Lubrizol (Wickliffe, OH, USA). A measure of 10 g of fresh inactive catalyst and 47.6 g of Sulfurzol were introduced to the autoclave reactor, and then the reactor was hermetically closed. Subsequently, the pressure test was performed at 140 bar for 20 min under a nitrogen atmosphere to identify possible leaks. After the pressure test, nitrogen was released, and the reactor was pressurized to the initial pressure of 50 bar by hydrogen; then, the temperature was increased to 340 °C (8.3 °C/min), and catalysts were activated at 340 °C for 60 min under a constant stirring of 500 rpm. After activation, the autoclave was cooled down to room temperature, and activated catalysts were dried at room temperature overnight and ground to obtain a homogeneous powder catalyst. A measure of 5 g of the activated catalyst and 50 g of the feedstock were introduced to the autoclave vessel, and the reactor was closed entirely. The pressure test was performed before the reaction to identify possible leaks, similar to the activation step. Then, nitrogen was released, and the feed gas switched to hydrogen, and the reactor was pressurized to 50 bar with hydrogen, and temperature increased to 450 °C with a heating rate of 8.3 °C/min under constant stirring (500 rpm). The hydrocracking reaction using the activated catalysts were performed at 450 °C, and the initial pressure of 50 bar under the constant stirring of 500 rpm. In total, 24 reactions were carried out in this study, using four different feedstocks, two different reaction times (60 and 120 min), and three different catalysts (Table 7).

### 3.3. Products Analysis

All gaseous and liquid products of the hydrocracking reaction were collected and analyzed using different techniques. The mass balance of the products was calculated by the weight of the total sediment and liquid filtrated (cold filtration). The density of the liquid products was measured at 15 °C using a semi-hydrometer KYOTO DA-645 (Kyoto Electronics Manufacturing co., Kyoto, Japan). The simulated distillation (SIMDIS) analysis was used to determine the boiling point distribution of the raw materials and products of the hydrocracking. The SIMDIS analysis was performed using a high-temperature gas chromatography, according to the standard test method ASTM D7169 [33]. An Agilent 7890 HT/SIMDIS system (Santa Clara, CA, USA) was used for this analysis, and the installed column was DBHT-SIMD, 5 m, 0.53 mm, 0.15 µm.

The sample was injected onto a gas chromatography column to separate the hydrocarbons by boiling points for this test. The gross and net calorific values of the liquid products were determined by VUHU (Vyzkumny Ustav Hnedeho Uhli, Most, Czech Republic) using a bomb calorimeter Parr 6300, delivered by Parr Instruments Company according to CSN ISO 1928 and CSN DIN 51900 norms [34,35]. The elemental analyzer FLASH 2000 was used to determine the carbon and hydrogen content of the hydrocracking products using the ASTM D5291 [36]. The sulfur and nitrogen contents of the products in ppm level were determined using Trace SN Cube Instrument (Elementar, Frankfurt, Germany), according to the standard test methods ASTM D5453 [37] and ASTM D4629 [38].

Gaseous products were analyzed using Agilent’s “Refinery Gas Analysis” method. The Agilent 7890A gas chromatograph has three channels: (i) a HayeSep Q column with a thermal conductivity detector (TCD) to measure H_2_ (N_2_ carrier gas); (ii) a HayeSep Q column with a TCD to measure O_2_, N_2_, CO, CO_2_, SH_2_, and C_1_–C_2_ hydrocarbons (He carrier gas); and (iii) a 5A molecular sieve column with a flame ionization detector to measure C_1_–C_7_ hydrocarbons (He carrier gas).

To evaluate the efficiency of hydrocracking reactions, the conversion and selectivity of different fractions, including gasoline, diesel, and jet fuel, were determined according to the Equations (1)–(6) [29,39]. The yield of each fraction was also obtained according to Equation (7).

(1) $$\mathrm{Conversion}\;\left(\mathrm{gasoline}\right)=\frac{{\mathrm{Feed}}_{>180{}^{\circ}C}-{\;\mathrm{Product}}_{>180{}^{\circ}C}}{{\mathrm{Feed}}_{>180{}^{\circ}C}}$$

(2) $$\mathrm{Gasoline}\;\mathrm{Selectivity}=\frac{{\mathrm{Product}}_{50-180{}^{\circ}C}-{\;\mathrm{Feed}}_{50-180{}^{\circ}C}}{{\mathrm{Feed}}_{>180{}^{\circ}C}-{\;\mathrm{Product}}_{>180{}^{\circ}C}}$$

(3) $$\mathrm{Conversion}\;\left(\mathrm{diesel}\right)=\frac{{\mathrm{Feed}}_{>360{}^{\circ}C}-{\;\mathrm{Product}}_{>360{}^{\circ}C}}{}$$

(4) $$\mathrm{Diesel}\;\mathrm{Selectivity}=\frac{{\mathrm{Product}}_{180-360{}^{\circ}C}-{\;\mathrm{Feed}}_{180-360{}^{\circ}C}}{{\mathrm{Feed}}_{>360{}^{\circ}C}-{\;\mathrm{Product}}_{>360{}^{\circ}C}}$$

(5) $$\mathrm{Conversion}\;\left(\mathrm{jet}\;\mathrm{fuel}\right)=\frac{{\mathrm{Feed}}_{>290{}^{\circ}C}-{\;\mathrm{Product}}_{>290{}^{\circ}C}}{{\mathrm{Feed}}_{>290{}^{\circ}C}}$$

(6) $$\mathrm{Jet}\;\mathrm{Fuel}\;\mathrm{Selectivity}=\frac{{\mathrm{Product}}_{120-290{}^{\circ}C}-{\;\mathrm{Feed}}_{120-290{}^{\circ}C}}{{\mathrm{Feed}}_{>290{}^{\circ}C}-{\;\mathrm{Product}}_{>290{}^{\circ}C}}$$

(7) Fraction Yield=Fraction Conversion × Fraction Selectivity

## 4. Conclusions

This work studied the performance of metal-transition phonolite-based catalysts (5Ni10W/Ph 5Ni10Mo/Ph and 5Co10Mo/Ph) for the hydrocracking of Fischer–Tropsch heavy fraction (i.e., waxes of distillation residues) and its blends with vacuum gas oil at 450 °C, under 50 bar. From the obtained results, it can be concluded that the 5Ni10W/A-Ph catalyst was more efficient for the hydrocracking of the feedstocks with higher FT residue wax, which are mainly linear paraffins and can be cracked easily, while the Mo-based catalysts (5Ni10Mo/A-Ph, and 5Co10Mo/A-Ph), as hydrotreating catalysts, showed a better performance for the hydrocracking of the feedstocks with higher VGO content with more polar and aromatic compounds, which need to be hydrotreated. Our article proved the suitability of our phonolite-based catalysts for FT residue and VGO blends processing. The preliminary evaluation covered different feedstock blends and two reaction times. This research gives essential information about the high potential of using modified phonolite as a support to produce an active catalyst for the VGO hydrocracking and many other similar reactions. The information given in this work enables the opportunity to optimize the overall hydrocracking system not only for fossil VGO fuel, but also for renewable feedstocks using near-available raw material to synthesize a phonolite modified catalyst.

## Figures and Tables

**Table 1:** The mass balance and heating values of the hydrocracking products at different reaction conditions.

Reaction	Reaction Conditions			Yields of Hydrocracking Products (wt.%)			Heating Value	
	Cat.	Feedstock	Time (min)	Gases	Liquids	Solid + Loss	Gross cal. val. (MJ/kg)	Net cal. val. (MJ/kg)
1	Ni-W	FT residue	60	16	68	16	44.61	41.48
2	Ni-Mo	FT residue	60	18	61	21	45.21	42.09
3	Co-Mo	FT residue	60	16	66	17	45.26	42.31
4	Ni-W	FT residue	120	24	58	18	45.28	42.28
5	Ni-Mo	FT residue	120	22	59	19	45.16	42.32
6	Co-Mo	FT residue	120	25	55	21	43.93	41.16
7	Ni-W	FT res.:VGO (9:1)	60	12	41	47	44.84	41.79
8	Ni-Mo	FT res.:VGO (9:1)	60	11	32	57	41.82	38.58
9	Co-Mo	FT res.:VGO (9:1)	60	13	64	24	45.54	42.37
10	Ni-W	FT res.:VGO (9:1)	120	19	57	24	46.04	42.93
11	Ni-Mo	FT res.:VGO (9:1)	120	23	58	19	46.62	43.50
12	Co-Mo	FT res.:VGO (9:1)	120	19	59	22	46.28	43.28
13	Ni-W	FT res.:VGO (7:3)	60	12	18	70	44.20	41.36
14	Ni-Mo	FT res.:VGO (7:3)	60	13	60	27	44.69	41.56
15	Co-Mo	FT res.:VGO (7:3)	60	14	69	17	43.87	40.82
16	Ni-W	FT res.:VGO (7:3)	120	22	58	20	45.78	42.89
17	Ni-Mo	FT res.:VGO (7:3)	120	18	60	22	45.14	42.11
18	Co-Mo	FT res.:VGO (7:3)	120	22	59	19	43.67	40.77
19	Ni-W	FT res.:VGO (5:5)	60	15	48	37	43.81	40.96
20	Ni-Mo	FT res.:VGO (5:5)	60	13	71	16	43.78	41.79
21	Co-Mo	FT res.:VGO (5:5)	60	15	61	25	44.51	41.60
22	Ni-W	FT res.:VGO (5:5)	120	19	61	20	42.87	40.06
23	Ni-Mo	FT res.:VGO (5:5)	120	21	59	21	44.00	41.17
24	Co-Mo	FT res.:VGO (5:5)	120	23	54	23	41.85	39.27

**Table 2:** Properties of the hydrocracking liquid products at different reaction conditions.

Reaction	Reaction Conditions			Product Composition and Properties						
	Cat.	Feedstock	Time (min)	C (%)	H (%)	S (ppm)	N (ppm)	H/C ratio	Density (15 °C)	Ref. Index ^1^ (20 °C)
1	Ni-W	FT residue	60	85.7	14.6	1080	5.22	2.03	772.10	1.4288
2	Ni-Mo	FT residue	60	87.5	13.7	1970	17.5	1.87	786.79	1.4385
3	Co-Mo	FT residue	60	85.4	14.1	1085	8.92	1.97	774.75	1.4298
4	Ni-W	FT residue	120	85.9	14.3	1852	6.84	1.98	765.39	1.4262
5	Ni-Mo	FT residue	120	85.9	14.3	1169	9.08	1.98	763.64	1.4258
6	Co-Mo	FT residue	120	86.1	13.8	2255	14.8	1.91	778.82	1.4333
7	Ni-W	FT res.:VGO (9:1)	60	85.5	14.5	1266	62.1	2.02	786.48	1.4400
8	Ni-Mo	FT res.:VGO (9:1)	60	85.5	14.0	2083	108	1.95	800.49	1.4471
9	Co-Mo	FT res.:VGO (9:1)	60	84.6	14.0	1839	95.6	1.97	790.99	1.4395
10	Ni-W	FT res.:VGO (9:1)	120	85.0	14.1	1697	78.7	1.98	779.30	1.4353
11	Ni-Mo	FT res.:VGO (9:1)	120	85.6	13.8	1536	78.8	1.92	775.42	1.4318
12	Co-Mo	FT res.:VGO (9:1)	120	85.7	14.3	1913	105	1.99	782.51	1.4364
13	Ni-W	FT res.:VGO (7:3)	60	85.6	13.8	2656	284	1.92	819.55	1.4472
14	Ni-Mo	FT res.:VGO (7:3)	60	86.3	13.6	2519	270	1.88	808.99	1.4527
15	Co-Mo	FT res.:VGO (7:3)	60	85.5	13.8	4390	274	1.92	806.82	1.4508
16	Ni-W	FT res.:VGO (7:3)	120	85.5	13.8	2041	233	1.92	795.20	1.4463
17	Ni-Mo	FT res.:VGO (7:3)	120	85.8	13.7	2358	215	1.90	800.47	1.4490
18	Co-Mo	FT res.:VGO (7:3)	120	85.6	13.9	2852	300	1.93	800.74	1.4479
19	Ni-W	FT res.:VGO (5:5)	60	86.7	13.7	3004	404	1.88	835.21	1.4700
20	Ni-Mo	FT res.:VGO (5:5)	60	86.5	13.7	3415	425	1.89	819.45	1.4600
21	Co-Mo	FT res.:VGO (5:5)	60	86.7	13.1	4612	325	1.80	817.72	1.4587
22	Ni-W	FT res.:VGO (5:5)	120	86.4	13.3	3738	340	1.83	816.33	1.4606
23	Ni-Mo	FT res.:VGO (5:5)	120	86.6	13.4	4023	435	1.84	823.78	1.4647
24	Co-Mo	FT res.:VGO (5:5)	120	87.2	12.8	6273	328	1.75	834.05	1.4720

^1^ Refractive index.

**Table 3:** Composition of the gaseous products of hydrocracking reaction.

Reaction	C_1_	C_2_–C_4_				C_5_			C_5+_	H_2_S	H_2_
		Alkane	Isoalkane	Alkene	Isoalkene	Alkane	Isoalkane	Alkene			
1	8.04	60.69	1.74	1.61	0.08	2.57	0.36	0.25	0.53	11.4	12.69
2	13.71	61.59	1.27	4.45	0.2	3.59	0.39	0.85	1.64	7.41	4.9
3	8.03	60.9	1.82	2.28	0.12	2.93	0.46	0.43	0.91	10.48	11.67
4	10.81	63.55	1.59	2.88	0.14	1.79	0.28	0.26	0.39	11.18	7.14
5	9.56	65.55	1.73	2.7	0.12	2.25	0.32	0.32	0.6	8.45	8.4
6	14.57	58.17	1.36	2.67	0.13	1.8	0.28	0.31	0.58	11.82	8.29
7	8.52	58.27	2.06	0.97	0.07	2.83	0.52	0.19	0.74	11.45	14.38
8	10.59	44.28	2.37	1.23	0.11	2.62	0.38	0.24	0.8	20.64	16.73
9	10.21	52.82	1.8	1.78	0.1	2.48	0.48	0.37	0.82	13.74	15.41
10	13.6	57.31	1.51	2.32	0.1	2.52	0.39	0.4	1.29	9.82	10.74
11	13.31	56.96	1.5	1.96	0.1	2.53	0.38	0.33	0.79	10.67	11.49
12	12.2	61.52	1.47	2.61	0.13	2.02	0.33	0.28	0.63	9.19	9.6
13	13.57	44.59	1.94	0.9	0.08	3.2	0.7	0.26	1.08	15.84	17.83
14	14.84	48.74	2.35	1.33	0.12	2.22	0.61	0.27	0.73	15.05	13.72
15	14.56	47.73	1.93	1.72	0.13	1.87	0.51	0.32	0.57	18.31	12.33
16	16.31	55.39	1.73	1.8	0.14	2.09	0.46	0.26	0.52	11.04	10.26
17	16.85	52.45	2.43	1.62	0.15	1.68	0.45	0.21	0.42	14.49	9.29
18	16.26	55.04	1.77	1.98	0.16	2.2	0.5	0.31	0.56	10.52	10.71
19	14.55	52.46	2.91	1	0.13	1.44	0.67	0.14	0.26	15.09	11.33
20	15.91	45.51	2.6	1.01	0.11	2.74	0.86	0.27	1.04	16.45	13.48
21	14.04	50.11	3.14	1.51	0.17	2.49	0.91	0.34	0.79	15.7	10.79
22	18.97	48.92	2.64	1.25	0.13	0.74	0.35	0.08	0.06	14.96	11.89
23	20.27	51.24	1.47	1.96	0.12	2.14	0.58	0.23	0.69	10.92	10.36
24	17.91	56.75	2.08	2.49	0.17	1.66	0.6	0.24	0.38	11.95	5.77

**Table 4:** Conversion, selectivity, and yield of hydrocracking standard products.

Reaction	Gasoline Fraction			Diesel Fraction			Jet Fuel Fraction		
	Conversion (%)	Selectivity (%)	Yield (%)	Conversion (%)	Selectivity (%)	Yield (%)	Conversion (%)	Selectivity (%)	Yield (%)
1	57.89	51.96	15.04	90.02	34.39	15.48	80.45	49.54	19.93
2	64.66	46.40	15.00	93.88	29.72	13.95	86.00	45.75	19.67
3	61.32	50.00	15.33	91.06	32.93	14.99	82.48	47.78	19.71
4	73.36	46.69	17.13	96.76	22.64	10.95	91.75	40.79	18.71
5	70.12	47.25	16.57	96.02	25.49	12.24	89.61	42.78	19.16
6	74.07	44.78	16.58	96.92	22.02	10.67	92.16	39.91	18.39
7	69.09	19.45	6.72	91.51	20.53	9.39	84.32	33.47	14.11
8	71.52	9.83	3.52	91.49	12.05	5.51	84.53	22.05	9.32
9	54.44	43.78	11.92	86.72	33.92	14.71	76.17	46.94	17.88
10	65.34	42.44	13.86	94.03	26.86	12.63	86.77	45.03	19.54
11	65.29	44.30	14.46	93.23	26.29	12.25	85.86	43.42	18.64
12	67.62	45.97	15.54	94.55	24.71	11.68	87.70	42.83	18.78
13	84.18	4.64	1.95	94.90	3.59	1.70	91.21	10.92	4.98
14	54.91	36.23	9.95	86.30	30.85	13.31	87.40	38.70	16.91
15	52.20	50.88	13.28	85.98	34.01	14.62	74.96	51.62	19.35
16	65.56	43.77	14.35	93.07	23.43	10.90	85.97	43.03	18.49
17	59.15	43.44	12.85	92.12	30.21	13.91	92.75	46.17	21.41
18	64.88	45.11	14.63	92.93	24.11	11.20	85.04	42.83	18.21
19	59.63	17.58	5.24	88.22	26.72	11.79	70.64	31.17	11.01
20	49.31	50.62	12.48	84.23	34.22	14.41	72.70	51.49	18.72
21	57.66	37.28	10.75	79.51	27.72	11.02	77.20	41.34	15.96
22	57.39	43.60	12.51	91.64	29.63	13.58	81.74	52.13	21.30
23	62.51	43.43	13.57	91.29	23.06	10.52	83.19	44.28	18.42
24	66.44	39.69	13.19	85.19	20.27	8.64	85.02	40.18	17.08

**Table 5:** Characteristics of the raw materials and feedstocks for the hydrocracking reaction.

Material	FT wax	FT res.	VGO	FT res. 9:1 VGO	FT res. 7:3 VGO	FT res. 5:5 VGO
C, %	84.8	84.5	84.9	86	86.1	86.3
H, %	14.8	14.7	9.94	14.2	13.9	13.6
N, ppm	102	83.2	847	184	330	528
S, ppm	8.45	29.6	19200	1880	5465	8891
H/C ratio	2.08	2.07	1.40	1.97	1.92	1.88
IBP-FBP (SIMDIS), °C	249–639	356–713	284–510	320–622	306–701	299–690

**Table 6:** XRD analysis of the utilized support material (A-Ph) and created catalysts. Textural properties of a phonolite-derived solids by Hg porosimetry and N_2_ physisorption [31].

Sample	A-Ph	5Ni10W/A-Ph	5Ni10Mo/A-Ph	5Co10Mo/A-Ph
Specific surface BET, m^2^/g	120.1	53.9	41.8	51.3
Hg porosimetry/Total pore volume, cm^3^/g	0.030	0.016	0	0
Total intrusion volume, m^2^/g	0.179	0.118	0.200	0.209
Si, wt.%	34.8	28.5	28.9	29.6
Al, wt.%	6.7	5.3	5.5	5.7
Ni, wt.%	0.0	5.2	5.4	0.0
W, wt.%	0.0	9.8	0.0	0.0
Mo, wt.%	0.0	0.0	10.0	9.0
Co, wt.%	0.0	0.0	0.0	4.6
Na, wt.%	2.8	1.7	1.2	1.4
K, wt.%	6.6	5.2	2.8	2.7
Fe, wt.%	0.8	0.5	0.6	0.6
Others, wt.%	<0.1	0.5	0.2	0.2
*O, wt.%	48.3	43.3	45.4	46.2

*Oxygen content calculated by difference.

**Table 7:** Different reaction conditions for the hydrocracking reaction.

Reaction	Reaction Conditions		
	Catalyst	Feedstock	Reaction Time (min)
1	5Ni10W/A-Ph	FT residue	60
2	5Ni10Mo/A-Ph	FT residue	60
3	5Co10Mo/A-Ph	FT residue	60
4	5Ni10W/A-Ph	FT residue	120
5	5Ni10Mo/A-Ph	FT residue	120
6	5Co10Mo/A-Ph	FT residue	120
7	5Ni10W/A-Ph	FT res.:VGO (9:1)	60
8	5Ni10Mo/A-Ph	FT res.:VGO (9:1)	60
9	5Co10Mo/A-Ph	FT res.:VGO (9:1)	60
10	5Ni10W/A-Ph	FT res.:VGO (9:1)	120
11	5Ni10Mo/A-Ph	FT res.:VGO (9:1)	120
12	5Co10Mo/A-Ph	FT res.:VGO (9:1)	120
13	5Ni10W/A-Ph	FT res.:VGO (7:3)	60
14	5Ni10Mo/A-Ph	FT res.:VGO (7:3)	60
15	5Co10Mo/A-Ph	FT res.:VGO (7:3)	60
16	5Ni10W/A-Ph	FT res.:VGO (7:3)	120
17	5Ni10Mo/A-Ph	FT res.:VGO (7:3)	120
18	5Co10Mo/A-Ph	FT res.:VGO (7:3)	120
19	5Ni10W/A-Ph	FT res.:VGO (5:5)	60
20	5Ni10Mo/A-Ph	FT res.:VGO (5:5)	60
21	5Co10Mo/A-Ph	FT res.:VGO (5:5)	60
22	5Ni10W/A-Ph	FT res.:VGO (5:5)	120
23	5Ni10Mo/A-Ph	FT res.:VGO (5:5)	120
24	5Co10Mo/A-Ph	FT res.:VGO (5:5)	120

## Data Availability

Not applicable.

## Sample Availability

Samples of the compounds are not available from the authors.
